# Screen-Printed Electrode Modified by Bismuth /Fe_3_O_4_ Nanoparticle/Ionic Liquid Composite Using Internal Standard Normalization for Accurate Determination of Cd(II) in Soil

**DOI:** 10.3390/s18010006

**Published:** 2017-12-21

**Authors:** Hui Wang, Guo Zhao, Yuan Yin, Zhiqiang Wang, Gang Liu

**Affiliations:** 1 Key Lab of Modern Precision Agriculture System Integration Research, Ministry of Education of China, China Agricultural University, Beijing 100083, China; wanghuilunwen@cau.edu.cn (H.W.); guozhao1989@gmail.com (G.Z.); yinyuan@cau.edu.cn (Y.Y.); 2 Key Lab of Agricultural Information Acquisition Technology, Ministry of Agricultural of China, China Agricultural University, Beijing 100083, China; 3 College of Computer Science and Technology, Shandong University of Technology, Zibo 255049, China; wzq@sdut.edu.cn

**Keywords:** electrochemistry, screen-printed electrode, bismuth, Fe_3_O_4_ nanoparticle, cadmium

## Abstract

The quality and safety of agricultural products are threatened by heavy metal ions in soil, which can be absorbed by the crops, and then accumulated in the human body through the food chain. In this paper, we report a low-cost and easy-to-use screen-printed electrode (SPE) for cadmium ion (Cd(II)) detection based on differential pulse voltammetry (DPV), which decorated with ionic liquid (IL), magnetite nanoparticle (Fe_3_O_4_), and deposited a bismuth film (Bi). The characteristics of Bi/Fe_3_O_4_/ILSPE were investigated using scanning electron microscopy, cyclic voltammetry, impedance spectroscopy, and linear sweep voltammetry. We found that the sensitivity of SPE was improved dramatically after functionalized with Bi/Fe_3_O_4_/IL. Under optimized conditions, the concentrations of Cd(II) are linear with current responses in a range from 0.5 to 40 µg/L with the lowest detection limit of 0.05 µg/L (S/N = 3). Additionally, the internal standard normalization (ISN) was used to process the response signals of Bi/Fe_3_O_4_/ILSPE and established a new linear equation. For detecting three different Cd(II) concentrations, the root-mean-square error using ISN (0.25) is lower than linear method (0.36). Finally, the proposed electrode was applied to trace Cd(II) in soil samples with the recovery in the range from 91.77 to 107.83%.

## 1. Introduction

Rice is one of the most popular staple food for large parts of Asia, especially in the south of China [1]. It is widely cultivated in these areas, which has the potential to improve the food production [2,3]. Due to unreasonable use of chemical fertilizer and sewage irrigation, cadmium, one of the most toxic heavy metals, has been deposited and exceeded the safety level in soil. Cadmium ions (Cd(II)) in soil are easily absorbed by the paddy [4], and then enter the human body through the digestive system [5,6,7,8]. Cd(II) can cause some serious diseases, such as carcinogenic issues, lung damage, kidney failure [7,9,10,11]. Therefore, quick and accurate identification of Cd(II) concentrations is urgent to prevent soil pollution and ensure food safety. Up to now, the regular methods have been employed for Cd(II) determination, such as X fluorescence spectrometry (XRF) [12], Atomic Absorption Spectroscopy (AAS) [13], Atomic Emission Spectrometry(AES) [14], Inductively Coupled Plasma-Atomic Emission Spectrometry (ICP-AES) [15], Inductively Coupled Plasma-Mass Spectrometry (ICP-MS) [16], Atomic Fluorescence Spectrometry (AFS) [17], biological technique [18] and electrochemical method [19]. However, the spectrographic methods are not suitable for in-situ analysis because of complex operation, large volume, and expensive instruments. The application of biological technique for metal ions detection was limited because the properties of biological materials hard to keep in natural conditions [20]. Compared to approaches mentioned above, the electrochemical method owns some unique merits such as relative portability, inexpensive instrumentation and real analysis [21]. It has been recognized as an effective way for heavy metal ions determination.

Screen-printed electrode (SPE) is low-cost and easy-to-use, which is much suited for mass production and application. Nevertheless, bare SPE is hard to trace Cd(II) at low concentrations. Many researches show that nanomaterials have merits to improve the performance of SPE. Magnetic nanoparticles (Fe_3_O_4_) [22,23,24] have broad applications in catalysis, biotechnology as well as analytical chemistry [25] due to the large surface area, quantum size effect, high adsorption ability, excellent biocompatibility, its strong superparamagnetic property, low toxicity and easy preparation. Srivastava et al. [26] developed an electrode coated ionic liquids and Fe_3_O_4_ nanoparticle, applied for the simultaneous determination of DNA bases.

Chitosan (CHT) is a natural polymer with abundant primary amino groups and hydroxyl groups. It has excellent adhesivity, hydrophilicity, gel-forming ability, doping feasibility, excellent mechanical stability, permeability, cost-effectiveness, and availability of reactive functional groups for chemical modifications. CHT has been widely employed in the construction of electrochemical sensors and biosensors to improve performances. It also can adsorb metal ions and various organic compounds at the same time.

To exploit a cheap, easy to use and sensitive electrode, we have developed a new electrode. It used a mixed solution of Fe_3_O_4_ and CHT to coat on the ion liquid based SPE (ILSPE), and then deposited a bismuth film (Bi) in situ. The new electrode can combine the advantages of Fe_3_O_4_, CHT, and SPE successfully. Additionally, Bi/Fe_3_O_4_/ILSPE was applied to determine Cd(II), and used internal standard normalization (ISN) to amendment the linear regression equation to improve the accuracy. At last, a new electroanalytical electrode for Cd(II) determination in soil was created.

## 2. Experimental

### 2.1. Chemicals and Reagents

All electrochemical measurements were collected using a CHI660D electrochemical workstation (CHI Instrument Company, Shanghai, China) with a three-electrode system. The modified screen-printed electrode was used as the working electrode, a platinum column as an auxiliary electrode and an Ag/AgCl electrode as a reference electrode. Scanning electron microscope photos were achieved by using the JEOLJEM-2011 device. All electrochemical measurements were recorded at room temperature.

Ionic liquid(IL), n-octylpyridinium hexafluorophosphate (OPFP), was purchased from Shanghai Chengjie Co., Ltd. (Shanghai, China). Graphite powder (size < 30 μm, 20019126) was obtained from Sinopham Chemical Reagent Co., Ltd. (Shanghai, China). SPE was bought from Suzhou delta system biological cross scientific research institute co. Ltd. (Shanghai, China). Chitosan and nano-Fe_3_O_4_ were obtained from Sigma-Aldrich Corporation (Shanghai, China). Standard solutions of Bi(III) and Cd(II) (1000 mg/L) were provided by National Standard Reference Materials Center of China and diluted as required. Phosphate buffer solution (0.2 M, pH 5.0) was selected as the supporting electrolyte for experiments. The rest of chemicals not mentioned here were of analytical reagent grade and were used as received. Millipore-Q (18.2 MΩ) water was used for all experiments.

### 2.2. Preparation of Fe_3_O_4_/ILSPE

**Figure 1:**
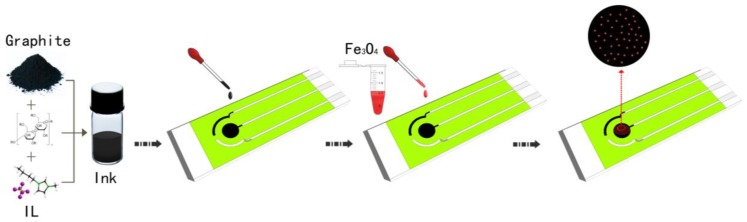
Fabrication and modification process.

The ILSPE was fabricated by the following steps in Figure 1. First, 0.05 g cellulose acetate was dissolved in a solution containing 2.5 mL cyclohexanone and 2.5 mL acetone. Second, 0.5 g OPPF and 2.0 g graphite powder were added and mixed to form a homogeneous and viscous ink achieved through ultrasonic shaking. The modified process was executed by using a pipette to direct write the composite onto the surface of SPE. Third, the prepared electrode was annealed at 80 °C for 30 min. 0.2 g chitosan flakes were weighed and dissolved in aqueous solution of 100 mL 1.0% acetic acid. Fourth, weighing 0.1 mg Fe_3_O_4_ nanoparticle was dispersed into 10 mL 0.2 wt % (weight/solution volume = 0.2 g/100 mL) CHT using an ultrasonic treatment for 2 h. 2.0 µL dispersion liquid was dropped on the ILSPE surface and dried at 50 °C for 30 min. For Bi(III) deposition, the 400 Bi(III) was added into the solution containing Cd(II), electrodeposited on Fe_3_O_4_/ILSPE at −1.2 V, and then reduced to Cd and Bi subsequently by DPV.

### 2.3. Sample Preparation

Soil samples were collected from an agriculture paddy field in Beijing, China, and pre-treated with the following steps. Briefly, soil samples were heated at 200 °C for 30 min in an oven to remove the water. The dried soil was ground using pestle and mortar, and sieved by a 200 μm sieve. 4 g soil sample was added to 40 mL of 0.1 M hydrochloric acid and extracted Cd(II) by ultrasonic processing for 60 min at room temperature. Finally, the supernatant was filtered by a membrane and adjusted to pH 5.0 using H_3_PO_4_ and NaH_2_PO_4_ solution.

### 2.4. Electrochemical Measurement Procedure

Differential pulse voltammetry (DPV) was applied to the detection of Cd(II) under optimized conditions. Cd(II) was performed in 0.2 mol/L phosphate buffer solution in the presence of 400 g/L Bi(III). Under stirring conditions, Cd(II) was deposited on the surface of the working electrode at the potential of −1.2 V for 240 s. After 20 s equilibration period, DPV was carried out from −1.2 V to 0 V with optimized parameters (increase E, 0.01 V; amplitude, 25 mV; pulse width, 0.2 s; sample width, 0.02 s; pulse period, 0.5 s).

## 3. Results

### 3.1. Characteristics

The morphological and electrochemical characteristics of the modified screen-printed electrode were investigated by some effective approaches.

**Figure 2:**
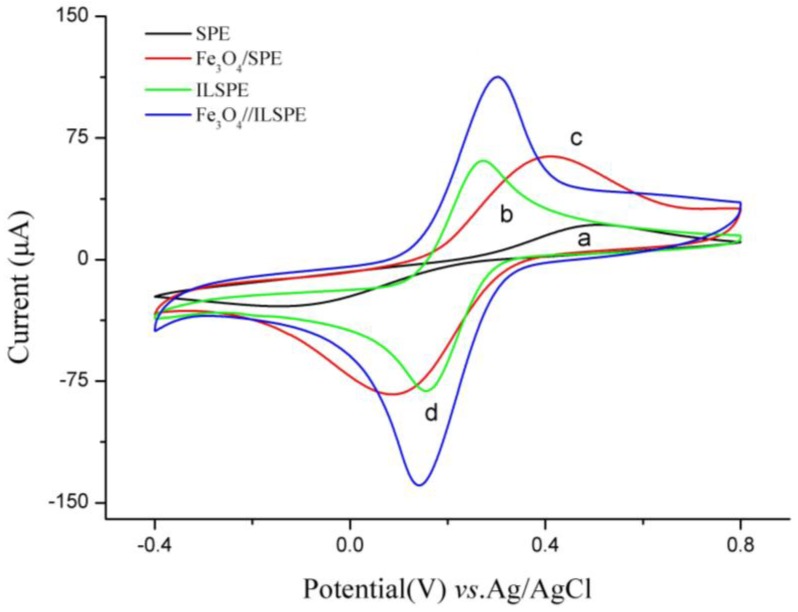
Cyclic voltammograms of different electrodes in 5 mM/L [Fe(CN)_6_]^3−/4−^ and 0.1 mol/L KCl, (**a**) SPE, (**b**) Fe_3_O_4_/SPE, (**c**) ILSPE, (**d**) Fe_3_O_4_/ILSPE. Scan rate: 50 mV/s.

Figure 2 shows the CV responses of different electrodes in a mixture of 5 mM [Fe(CN)_6_]^3−/4−^ and 0.1 M KCl. SPE (curve a) had a pair of weak oxidation peaks with a peak to peak separation of 380 mV. This indicated the electron transfer rate of SPE was sluggish due to the poor conductivity of commercial ink. While on ILSPE (curve b), the redox peak currents enhanced in view of IL. After modifying Fe_3_O_4_ nanoparticle (curve c), the peak currents increased considerably, demonstrating that nanomaterial membrane covered on SPE can improve the charge transfer [27]. One reason for these results may be the high specific surface area and excellent conductivity of Fe_3_O_4_ nanoparticle. Another reason that the Fe_3_O_4_ nanoparticle provides redox substances including ferrous and ferric iron. Well-defined reduction peaks were observed at the Fe_3_O_4_/ILSPE, whereas the peak potential separation reduced to 120 mV and the redox currents were considerably higher than those at the SPE, Fe_3_O_4_/SPE, and ILSPE due to the synergistic effects from ion liquid and Fe_3_O_4_ nanoparticles.

**Figure 3:**
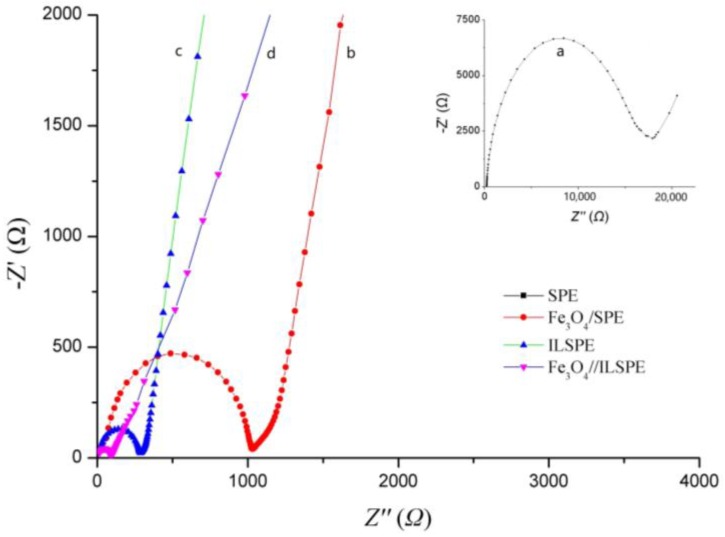
Electrochemical impedance spectra of different electrode in 5.0 mmol/L K_4_[Fe(CN)_6_] + 0.1 mol/L KCl solution with the frequencies from 100,000 to 1 Hz, Ω (**a**) SPE, (**b**) Fe_3_O_4_/SPE, (**c**) ILSPE, (**d**) Fe_3_O_4_/ILSPE.

Figure 3 shows the electron transfer kinetics of different electrodes were investigated by using EIS measurement. According to the Randle model, the value of electron-transfer resistance (*R*_ct_) is equal to the bulk membrane resistance coupled with the contact resistance. As shown in Figure 3b, the *R*_ct_ of SPE was got as 18 KΩ, indicating bare SPE had poor conductivity that went against electron-transfer. When SPE was modified by OPFP and Fe_3_O_4_ nanoparticle respectively, the electron transfer rate was improved, and impedance was decreased. The values of *R*_ct_ are 1025 Ω and 250 Ω for Fe_3_O_4_/SPE and ILSPE. After SPE modified OPFP and Fe_3_O_4_ nanoparticle at the same time, an obviously reduced impedance with the *R*_ct_ of 100 Ω was observed due to the synergistic effect of OPFP and Fe_3_O_4_ nanoparticles. These results are in agreement with the conclusion obtained from the CV.

**Figure 4:**
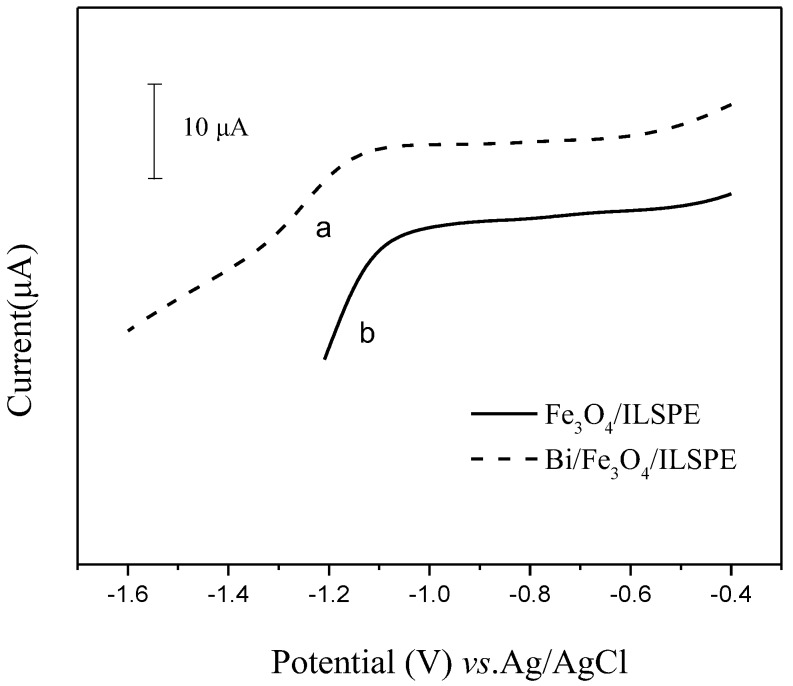
Linear sweep voltammograms in 0.2 mol/L phosphate buffer solution (**a**) Bi/Fe_3_O_4_/ILSPE, (**b**) Fe_3_O_4_/ILSPE.

The hydrogen evolution on the electrode surface diminishes the accuracy of the stripping analysis significantly. The linear sweep voltammetry (LSV) responses of different electrodes in 0.2 mol/L phosphate buffer solution (pH 5.0) were shown in Figure 4, respectively. The Fe_3_O_4_/ILSPE without bismuth film exhibited a relatively positive hydrogen overvoltage potential (about −1.1 V). While on Bi/Fe_3_O_4_/ILSPE, a more negative hydrogen evolution potential (about −1.2 V) due to the formation of bismuth film. It is reported that the bismuth electrodes are less prone to hydrogen evolution, due to the unique crystal plane structure of bismuth. This indicated that modified electrode has a sufficient potential window for the stripping analysis of Cd(II).

**Figure 5:**
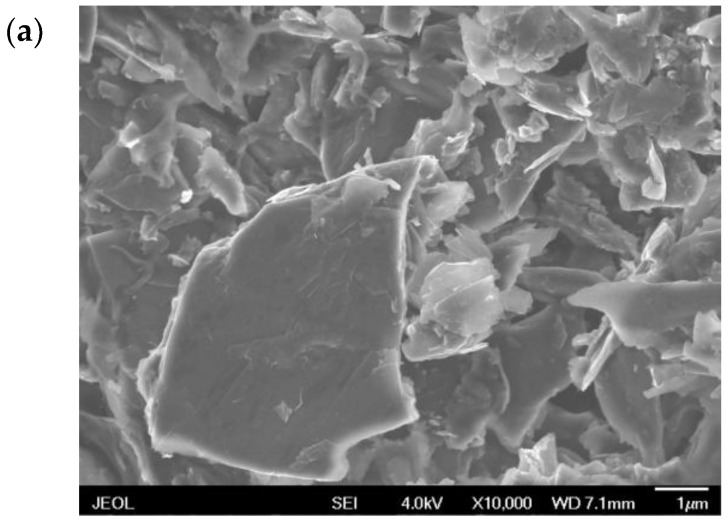

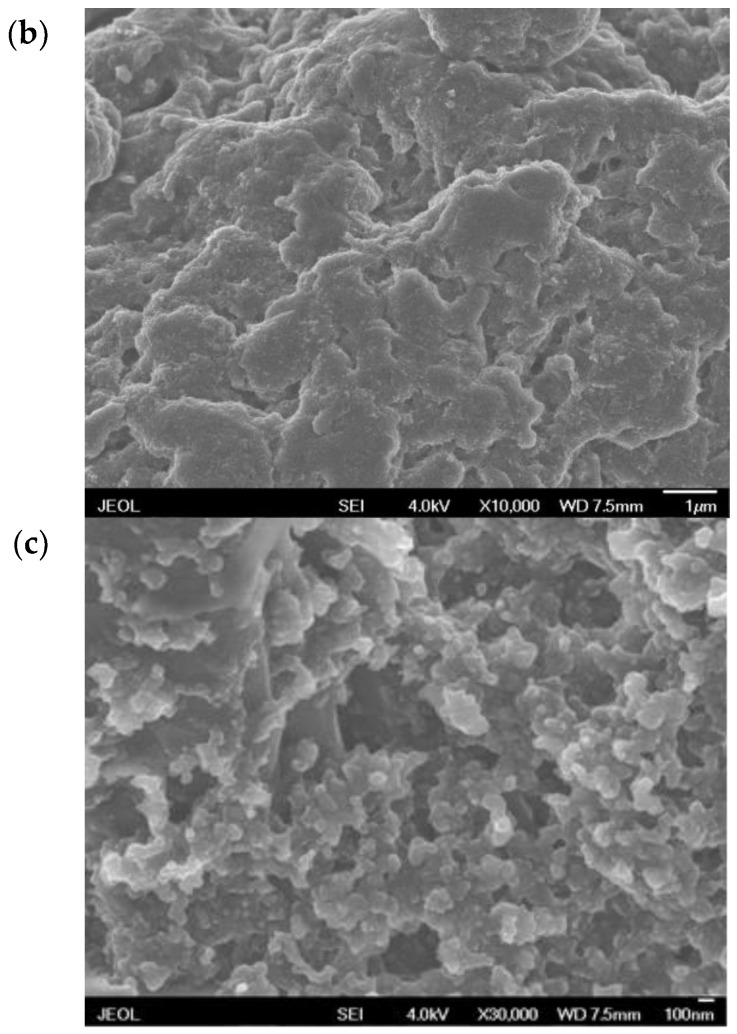
SEM images of (**a**) SPE, (**b**) ILSPE and (**c**) Fe_3_O_4_/ILSPE.

Scanning electron microscope (SEM) was used to research the surface morphological structures of prepared electrodes. As seen in Figure 5a, the surface of SPE shown a disordered distribution of gaps between graphite blocks, which affirmatively affected the conductivity of electrode. While on the ILSPE, the surface was compact and homogeneous (Figure 5b), indicating the solid binder OPFP has excellent adhesiveness to graphite particles in the view of the low melting point of OPFP. Figure 5c exhibited the typical SEM image of Fe_3_O_4_/ILSPE; it was observed that some ball-like structures covering the ILSPE’s surface, showing good interfacial interactions between Fe_3_O_4_ nanoparticles and ILSPE’s surface.

**Figure 6:**
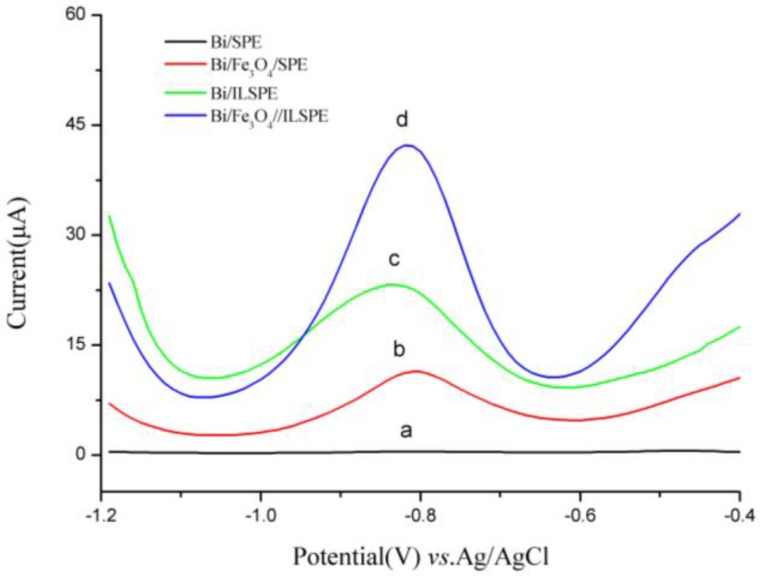
DPV responses of 40 µg/L Cd(II) and 400 µg/L Bi(III) in 0.2 mol/L phosphate buffer solution (pH 5.0) on the (**a**) SPE, (**b**) Fe_3_O_4_/SPE, (**c**) ILSPE, and (**d**) Fe_3_O_4_/ILSPE. Deposition time: 240 s. Deposition potential: −1.2 V.

The DPV responses of 40 µg/L Cd(II) on different electrodes are shown in Figure 6 to illustrate the sensitive improvement. A little peak current was observed on the SPE. After using Fe_3_O_4_ nanoparticles, Fe_3_O_4_/SPE exhibited higher stripping response toward Cd(II) determination. The main reason might be that the superior properties of Fe_3_O_4_ such as huge specific surface area and good conductivity decreased the electron transfer barrier, and accelerated the rate of metal ions preconcentration on the electrode surface. While on the ILSPE, the striping currents of Cd(II) is also enhanced in the view of the excellent conductivity and adhesion of ionic liquid. The highest stripping peak was observed on the Fe_3_O_4_/ILSPE, which might be attributed to IL and Fe_3_O_4_ composite film modification. This composite membrane not only enhanced the conductivity of the electrode but also offered abundant anchor sites for the detection of Cd(II).

### 3.2. Optimization of Experimental Parameters

**Figure 7:**
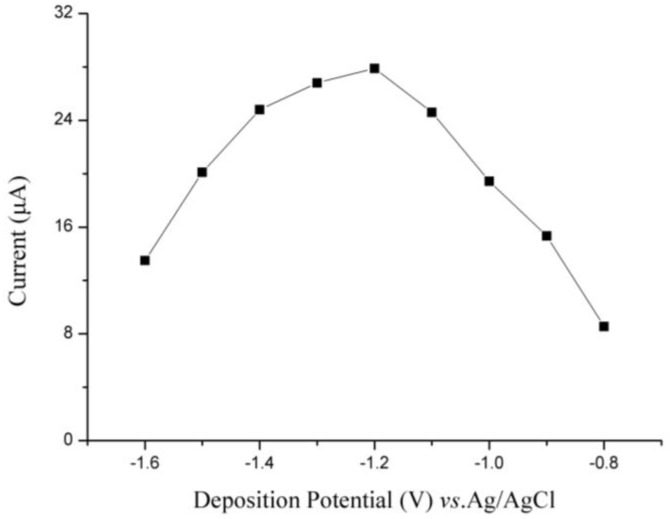
Effects of deposition potential on the stripping peaks current of 35 µg/L Cd(II).

The detecting parameters were optimized in 0.2 M phosphate buffer solution containing 35 µg/L of Cd(II). The relationship between the peak current of Bi/Fe_3_O_4_/ILSPE and deposition potential is described in Figure 7. With the deposition potential shifting from −0.8 V to −1.2 V, the peak current increased significantly, which the maximum value was achieved at −1.2 V. This may be the lower potential can offer more energy to improve the efficiency of accumulation. However, when the deposition potential is ranging from −1.2 V to −1.6 V, the peak currents decreased. We attribute this to the electrochemical potential stability window and interference from H_2_ evolution on the deposited Bi/Fe_3_O_4_/ILSPE surface. Thus, a deposition potential of −1.2 V was determined as optimum and employed in further experiments.

**Figure 8:**
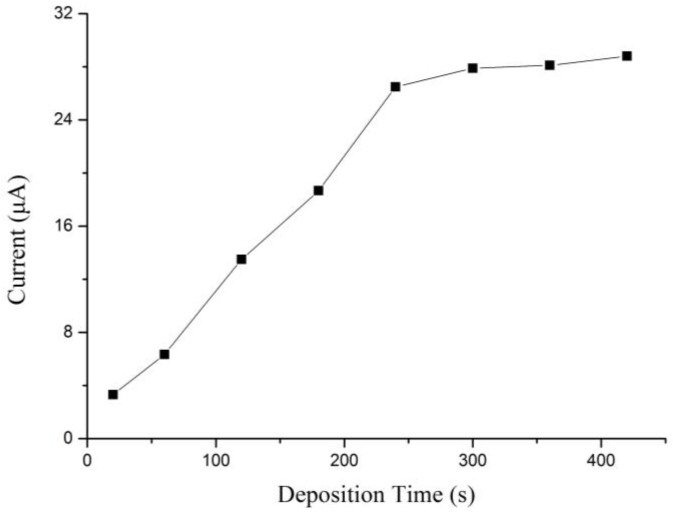
Effects of deposition time on the stripping peaks current of 35 µg/L Cd(II).

The peak current of Bi/Fe_3_O_4_/ILSPE changed along with deposition time is shown in Figure 8. The peak current increased proportionally with the prolonging of deposition time from 20 to 240 s. While over 240 s, the stripping current of the peak is not any more linear with deposition time, which is increasing slowly. In view both of sensitivity and determination time, a deposition time of 240 s was selected for further studies.

**Figure 9:**
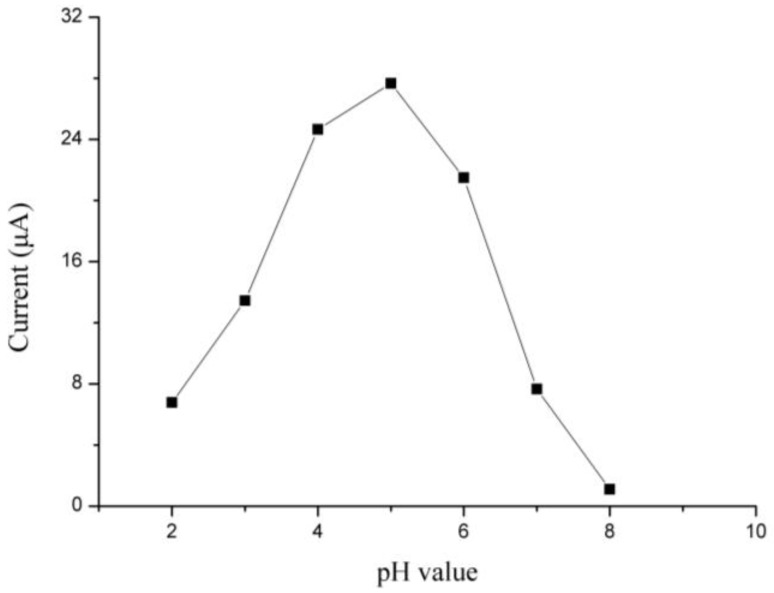
Effects of pH value on the stripping peaks current of 35 µg/L Cd(II).

Figure 9 shows the stripping response of Cd(II) on the Bi/Fe_3_O_4_/ILSPE affected with changing pH value. The peak current increased gradually with decreasing pH from 2.0 to 5.0, while the stripping response disappeared completely at high pH values. The maximum peak current was found at pH 5.0. At excessively acidic circumstances, enhanced hydrogen evolution will damage the quality of the Bi(III) film. If the pH is much higher, Bi(III) ions might be susceptible to hydrolysis in neutral and alkaline media. Thus, a pH of 5.0 was found to be suitable for experiments.

**Figure 10:**
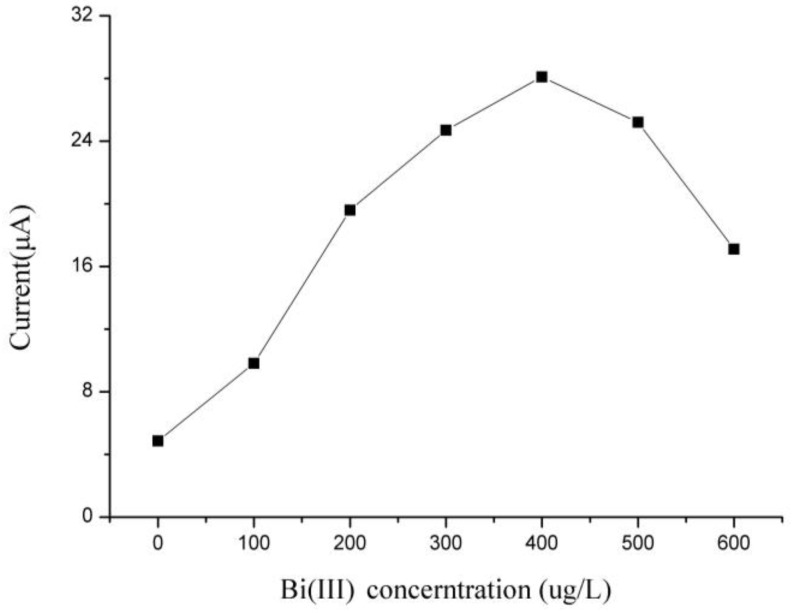
Effects of Bi(III) concentration on the stripping peaks current of 35 µg/L Cd(II).

Figure 10 shows the stripping response of Cd(II) on the Bi/Fe_3_O_4_/ILSPE affected by the Bi(III) concentration. The peak current of Cd(II) was positively correlated with different levels of Bi(III) from 100 to 400 µg/L and negatively correlated when the level of Bi(III) exceeded 400 µg/L. This may be attributed to the formation of thick bismuth layer on the electrode surface, which is not favorable for Cd(II) diffusing out. Consequently, 400 µg/L was selected as the optimal Bi(III) concentration.

**Figure 11:**
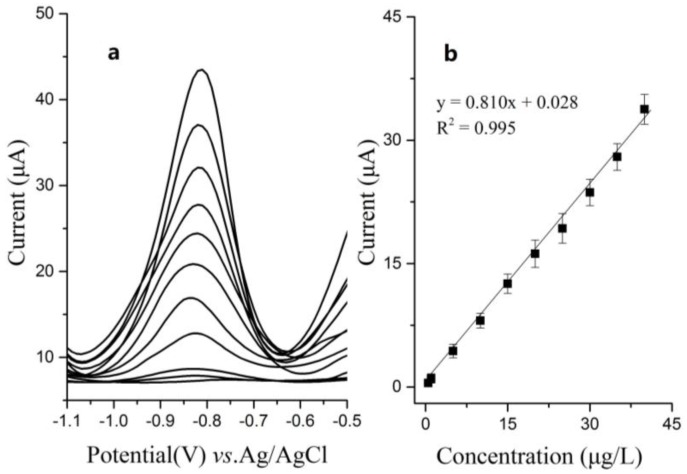
(**a**) Differential pulse voltammograms of Bi/Fe3O4/ILSPE for additions of 0, 0.5, 1, 5, 10, 15, 20, 25, 30, 35, 40 g/L Cd(II); (**b**) The right part shows the calibration curves. Error bar: n = 3.

The sensitivity of DPV is much higher than cyclic voltammograms. Figure 11 shows DPV responses of Bi/Fe_3_O_4_/ILSPE for different Cd(II) concentrations in 0.2 M phosphate buffer (pH 5.0). The deposition potential was kept at −1.2 V for the stripping analyses. Figure 11a shows different sharp stripping peaks on the modified electrode. As seen from the calibration curve in Figure 11a, a linear relationship was obtained between the peak current and Cd(II) concentration in the range from 0.5 µg/L to 40 µg/L with the detection limit as 0.05 µg/L (S/N = 3). The linear regression equation was I(µA) = 0.810 × *C*(µg/L) + 0.028 (r = 0.996), where I is the peak current and *C* is the concentration of Cd(II).

There are some differences in sensitivity among Bi/Fe_3_O_4_/ILSPEs, which will influence the accuracy of the results. To conquer these deficiencies, the internal standard normalization (ISN) was used to calibrate the linear regression equations. Following this method, we can get a new linear regression equation as *N* = 0.907 × *C*(µg/L) + 0.032(r = 0.996), where *N* is the ratio of 10 × I/I_0_, I_0_ is the response current of 10 µg/L, and *C* is the concentration of Cd(II). To demonstrate the improvement of measuring accuracy using this new method, we selected three electrodes to detect the different concentration of Cd(II) and obtained the detection value of I0 (E1: 0.93 µA; E2: 0.87 µA; 0.98 µA). As shown in Table 1, the results using ISN method are more closed to the real value, and relative error are much lower. Meanwhile, the root-mean-square errors of linear method and ISN are 0.36 and 0.25, respectively. This indicates that the ISN method can conquer parts of difference between electrodes and improve the measuring precision. root-mean-square error (0.257) is lower than linear method (0.36).

Compared with some published electrodes for Cd(II) detection [28,29,30,31], some properties are summarized in Table 2. The Bi/Fe_3_O_4_/ILSPE has a relatively wide linear detection range and low detection limit. Otherwise, the accumulation time is much shorter than others, which is far more suitable for rapid detection.

### 3.3. Interference Effects

**Figure 12:**
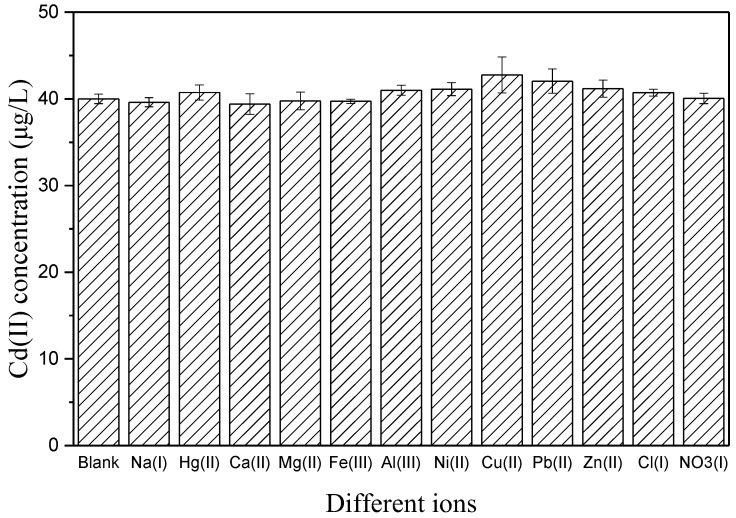
The 40 µg/L of Cd(II) before and after adding 50-fold concentrations of Na(I), Hg(II), Ca(II), Mg(II), Fe(III), Al(III), Ni(II), Cu(II), Pb(II), Zn(II) Cl(I), NO_3_(I). The error bars represent standard deviations based on three independent measurements.

The interference effected from various common-existed cations and anions in soil were a vital property for the electrochemical electrode in Figure 12. The effects of different interferences on the Bi/Fe_3_O_4_/ILSPE were estimated by DPV in phosphate buffer solution (pH 5.0) containing 40 µg/L of Cd(II) in the absence and presence a specified concentration of interfering substances and comparison of the peak currents. Under the optimized conditions, it was found that 50-fold concentrations of Na(I), Hg(II), Ca(II), Mg(II), Fe(III), Al(III), Ni(II), Cu(II), Pb(II), Zn(II) Cl(I), NO_3_(I), did not interfere with Cd(II) determination (the peak current change < 10%).

### 3.4. The Reproducibility

The reproducibility of the Bi/Fe_3_O_4_/ILSPE was evaluated using the two electrodes to determinate 10 µg/L Cd(II). The electrodes were stored at ambient conditions, which was used to measure the Cd(II) every seven days. The relative standard deviation (RSD) was 5.79% for Cd(II) in three-time measurements. The electrode lost less than 5.29% for Cd(II) of its original responses after its storage at ambient conditions for 14 days.

### 3.5. Application to Real Sample Analysis

To evaluate the practical performance, Bi/Fe_3_O_4_/ILSPE was applied to detect Cd(II) in the soil samples. Each extract solution undergoes three parallel determinations in Table 3. Cd(II) in soil samples could be satisfactorily detected with the recovery in the range from 91.77 to 107.83%. For comparing the results of the suggested method with those of FAAS, the *t*-test was applied. It can be concluded that there is no significant difference between the results obtained by the two methods for *p* > 0.05. All results indicated that the present Bi/Fe_3_O_4_/ILSPE electrode could be used for the accurate detection of Cd(II) in soil samples.

## 4. Conclusions

In summary, a low-cost and simple method was used to prepare bismuth film/Fe_3_O_4_/IL composite modified screen-printed electrode, which was further investigated by CV, EIS, SEM, LSV, and DPV. Compared with Bi/SPE, Bi/IL/SPE, and Bi/Fe_3_O_4_/SPE, the electrode exhibited some good properties that include excel

ent electrochemical activity and accelerated charge transfer kinetics, which owed to the presence of conductive Fe_3_O_4_/IL composite and the co-deposits ability with heavy metals of bismuth film. The electrode was used internal standard normalization (ISN) to conquer differences between electrodes, which was further confirmed by the real samples analysis with a good accuracy and satisfactory recovery results.

## Figures and Tables

**Table 1:** Determination results of different Cd(II) concentration.

Cd(II) Concentration (µg/L)	Electrode Number	Current (µA)	Linear Method		ISN Method	
			Detection Concentration (µg/L)	Relative Error (%)	Detection Concentration (µg/L)	Relative Error (%)
2	E1	1.78 ± 0.05	1.93 ± 0.05	3.52	2.09 ± 0.06	4.48
	E2	1.63 ± 0.03	1.76 ± 0.03	11.89	2.03 ± 0.04	1.65
	E3	1.73 ± 0.05	1.87 ± 0.05	6.49	1.92 ± 0.06	3.92
5	E1	4.33 ± 0.19	4.73 ± 0.22	5.32	5.13 ± 0.26	2.60
	E2	4.22 ± 0.09	4.61 ± 0.10	7.74	5.34 ± 0.12	6.80
	E3	4.47 ± 0.12	4.89 ± 0.14	2.23	4.99 ± 0.16	0.24
10	E1	8.72 ± 0.29	9.57 ± 0.31	4.30	10.30 ± 0.37	3.00
	E2	8.53 ± 0.19	9.25 ± 0.21	7.49	10.64 ± 0.27	6.40
	E3	9.73 ± 0.37	10.68±0.40	6.83	9.78 ± 0.46	2.20

**Table 2:** Comparison with other electrodes for Cd(II) detection.

Modifier	Accumulation Time (s)	Concentration Range (µg/L)	Detection Limit (µg/L)	Reference
Bi/SG/SPE	240	0.95–16.9	1.4	[28]
G/PANI/PS/SPE	180	10–500	4.43	[29]
SbSPCE	120	11.5–72.4	3.4	[30]
ILGPE	300	0.1–3.2	0.01	[31]
Bi/Fe_3_O_4_/ILSPE	240	0.5–40	0.05	The present

**Table 3:** Determination results of Cd(II) in soil samples.

Sample	Added (μg/L)	Found (μg/L)	Found by FAAS (μg/L)	Recovery (%)
1	–	1.45 ± 0.49	1.38 ± 0.11	–
	5	6.88 ± 0.26		107.83
	10	11.48 ± 0.45		100.87
2	–	2.85 ± 0.37	3.02 ± 0.08	–
	5	7.36 ± 0.39		91.77
	10	12.66 ± 0.19		97.23

## References

[1] Wu Y., Zhang H., Liu G., Zhang J., Wang J., Yu Y., Lu S. (2016). Concentrations and health risk assessment of trace elements in animal-derived food in southern China. Chemosphere.

[2] Liu F., Liu X., Ding C., Wu L. (2015). The dynamic simulation of rice growth parameters under cadmium stress with the assimilation of multi-period spectral indices and crop model. Field Crops Res..

[3] Nharingo T., Ndumo T., Moyo M. (2015). Human health risks due to heavy metals through consumption of wild mushrooms from Macheke forest, Rail Block forest and Muganyi communal lands in Zimbabwe. Environ. Monit. Assess..

[4] Wang M., Chen W., Peng C. (2016). Risk assessment of Cd polluted paddy soils in the industrial and township areas in Hunan, Southern China. Chemosphere.

[5] Ikeda M., Nakatsuka H., Watanabe T., Shimbo S. (2015). Estimation of daily cadmium intake from cadmium in blood or cadmium in urine. Environ. Health Prev. Med..

[6] Jallad K.N. (2015). Heavy metal exposure from ingesting rice and its related potential hazardous health risks to humans. Environ. Sci. Pollut. Res. Int..

[7] Oteef M.D., Fawy K.F., Abd-Rabboh H.S., Idris A.M. (2015). Levels of zinc, copper, cadmium, and lead in fruits and vegetables grown and consumed in Aseer Region, Saudi Arabia. Environ. Monit. Assess..

[8] Roy M., McDonald L.M. (2015). Metal Uptake in Plants and Health Risk Assessments in Metal-Contaminated Smelter Soils. Land Degrad. Dev..

[9] Khan M.U., Muhammad S., Malik R.N., Khan S.A., Tariq M. (2015). Heavy metals potential health risk assessment through consumption of wastewater irrigated wild plants: A case study. Hum. Ecol. Risk Assess. Int. J..

[10] Panhwar A.H., Kazi T.G., Afridi H.I., Arain S.A., Arain M.S., Brahaman K.D., Naeemullah, Arain S.S. (2016). Correlation of cadmium and aluminum in blood samples of kidney disorder patients with drinking water and tobacco smoking: Related health risk. Environ. Geochem. Health.

[11] Zhu P., Liang X.X., Wang P., Wang J., Gao Y.H., Hu S.G., Huang Q., Huang R., Jiang Q., Wu S.X. (2016). Assessment of dietary cadmium exposure: A cross-sectional study in rural areas of south China. Food Control.

[12] Sacristán D., Viscarra Rossel R.A., Recatalá L. (2016). Proximal sensing of Cu in soil and lettuce using portable X-ray fluorescence spectrometry. Geoderma.

[13] Peng Y.E., Guo W., Zhang P., Jin L., Hu S. (2015). Heated Slurry Sampling for the Determination of Cadmium in Food by Electrothermal Atomic Absorption Spectrometry. Anal. Lett..

[14] Zhao Y., Li Z., Ross A., Huang Z., Chang W., Ou-Yang K., Chen Y., Wu C. (2015). Determination of heavy metals in leather and fur by microwave plasma-atomic emission spectrometry. Spectrochim. Acta Part B At. Spectrosc..

[15] Zhao J., Yan X., Zhou T., Wang J., Li H., Zhang P., Ding H., Ding L. (2015). Multi-throughput dynamic microwave-assisted leaching coupled with inductively coupled plasma atomic emission spectrometry for heavy metal analysis in soil. J. Anal. At. Spectrom..

[16] Chen L., Yuan D., Zhang X., Lv X., Wang L., Li J. (2015). Elemental analysis of Acori Tatarinowii Rhizomaby inductively coupled plasma emission and mass spectrometry with chemometrics. Anal. Lett..

[17] Zhang Y., Mao X., Wang M., Gao C., Qi Y., Qian Y., Tang X., Zhou J. (2015). Direct Determination of Cadmium in Grain by Solid Sampling Electrothermal Vaporization Atomic Fluorescence Spectrometry with a Tungsten Coil Trap. Anal. Lett..

[18] Li Y., Chen W.C., Chen S.M., Lou B.S., Ali M.A., Al-Hemaid F.M. (2014). Detection of real sample DNA at a cadmium sulfide-chitosan/gelatin modified electrode. Colloids Surf. B Biointerfaces.

[19] Singh J., Huerta-Aguilar C.A., Singh H., Pandiyan T., Singh N. (2015). Voltammetric Simultaneous Determination of Cu^2+^, Cd^2+^ and Pb^2+^ in Full Aqueous Medium Using Organic Nanoparticles of Disulfide Based Receptor. Electroanalysis.

[20] Wang Z., Liu G., Zhang L., Wang H. (2013). Electrochemical detection of trace cadmium in soil using a Nafion/stannum film-modified molecular wire carbon paste electrodes. Ionics.

[21] Bayrak H.E., Bulut V.N., TÜFekÇİ M., Bayrak H., Duran C., Soylak M. (2016). Comparative study for the separation, preconcentration, and determination of copper and cadmium in real samples by using two different ligands. Turk. J. Chem..

[22] Benvidi A., Jahanbani S., Akbari A., Zare H.R. (2015). Simultaneous determination of hydrazine and hydroxylamine on a magnetic bar carbon paste electrode modified with reduced graphene oxide/Fe_3_O_4_ nanoparticles and a heterogeneous mediator. J. Electroanal. Chem..

[23] Dehdashtian S., Gholivand M.B., Shamsipur M., Kariminia S. (2016). Construction of a sensitive and selective sensor for morphine using chitosan coated Fe_3_O_4_ magnetic nanoparticle as a modifier. Mater. Sci. Eng. C Mater. Biol. Appl..

[24] Gu T., Wang J., Xia H., Wang S., Yu X. (2014). Direct Electrochemistry and Electrocatalysis of Horseradish Peroxidase Immobilized in a DNA/Chitosan-Fe_3_O_4_ Magnetic Nanoparticle Bio-Complex Film. Materials.

[25] Gao C., Yu X.Y., Xiong S.Q., Liu J.H., Huang X.J. (2013). Electrochemical detection of arsenic(III) completely free from noble metal: Fe_3_O_4_ microspheres-room temperature ionic liquid composite showing better performance than gold. Anal. Chem..

[26] Kaur B., Srivastava R. (2014). Ionic liquids coated Fe_3_O_4_ based inorganic–organic hybrid materials and their application in the simultaneous determination of DNA bases. Colloids Surf. B.

[27] Yu C.L., Lo N.C., Cheng H., Tsuda T., Sakamoto T., Chen Y.H., Kuwabata S., Chen P.Y. (2014). An ionic liquid-Fe_3_O_4_ nanoparticles-graphite composite electrode used for nonenzymatic electrochemical determination of hydrogen peroxide. J. Electroanal. Chem..

[28] Dimovasilis P.A., Prodromidis M.I. (2015). Preparation of Screen-Printed Compatible Bismuth-Modified Sol-Gel Microspheres: Application to the Stripping Voltammetric Determination of Lead and Cadmium. Anal. Lett..

[29] Promphet N., Rattanarat P., Rangkupan R., Chailapakul O., Rodthongkum N. (2015). An electrochemical sensor based on graphene/polyaniline/polystyrene nanoporous fibers modified electrode for simultaneous determination of lead and cadmium. Sens. Actuators B Chem..

[30] Sosa V., Barcelo C., Serrano N., Arino C., Diaz-Cruz J.M., Esteban M. (2015). Antimony film screen-printed carbon electrode for stripping analysis of Cd(II), Pb(II), and Cu(II) in natural samples. Anal. Chim. Acta.

[31] Zhang X., Zhang Y., Ding D., Zhao J., Liu J., Yang W., Qu K. (2016). On-site determination of Pb^2+^ and Cd^2+^ in seawater by double stripping voltammetry with bismuth-modified working electrodes. Microchem. J..

